# Purple Phototrophic Bacteria as a Novel Platform for Coenzyme Q10 Production from CO_2_, Syngas, and Sulfur Waste Byproducts

**DOI:** 10.1021/acs.est.6c07111

**Published:** 2026-10-22

**Authors:** Elisa Huang-Lin, Raquel Lebrero, Sara Cantera

**Affiliations:** † Department of Chemical Engineering and Environmental Technology, 16782 University of Valladolid, Dr. Mergelina s/n, Valladolid 47011, Spain; ‡ Institute of Sustainable Processes, University of Valladolid, Dr. Mergelina s/n, Valladolid 47011, Spain

**Keywords:** CO_2_ utilization, coenzyme Q10, purple phototrophic bacteria, sulfur byproduct valorization, syngas bioconversion

## Abstract

The efficient conversion of CO_2_ and industrial waste streams into value-added products remains a critical challenge for sustainable microbial processes. Here, we present the first systematic demonstration of purple phototrophic bacteria (PPB) as platforms for the autotrophic valorization of CO_2_ and industrial off-gases or sulfur byproducts into valuable metabolites. Genome-guided screening combined with experimental validation identified strains capable of coupling photoautotrophic growth with the production of CoQ10 and carotenoid pigments. Among them, *Rhodospirillum rubrum* DSM 467 and DSM 107 exhibited the highest performance, maintaining stable CO_2_ and H_2_ consumption under semicontinuous operation and accumulating up to 9.6 ± 0.2 mg_CoQ10_ g_biomass_^–1^ under CO_2_/ H_2_ conditions, comparable to engineered production systems. In addition, *R. rubrum* DSM 467 efficiently oxidized CO from syngas via the water–gas shift reaction, displaying hierarchical substrate utilization driven by energetic constraints. In contrast, *Marichromatium purpuratum* simultaneously oxidized H_2_ and thiosulfate (S_2_O_3_^2–^), enabling parallel electron input into the photosynthetic electron transport chain and enhanced growth. Overall, these results establish PPB as robust and metabolically flexible biocatalysts for converting CO_2_ and industrial waste streams into high-value chemicals with direct applications in the pharmaceutical and nutraceutical industries.

## Graphical abstract

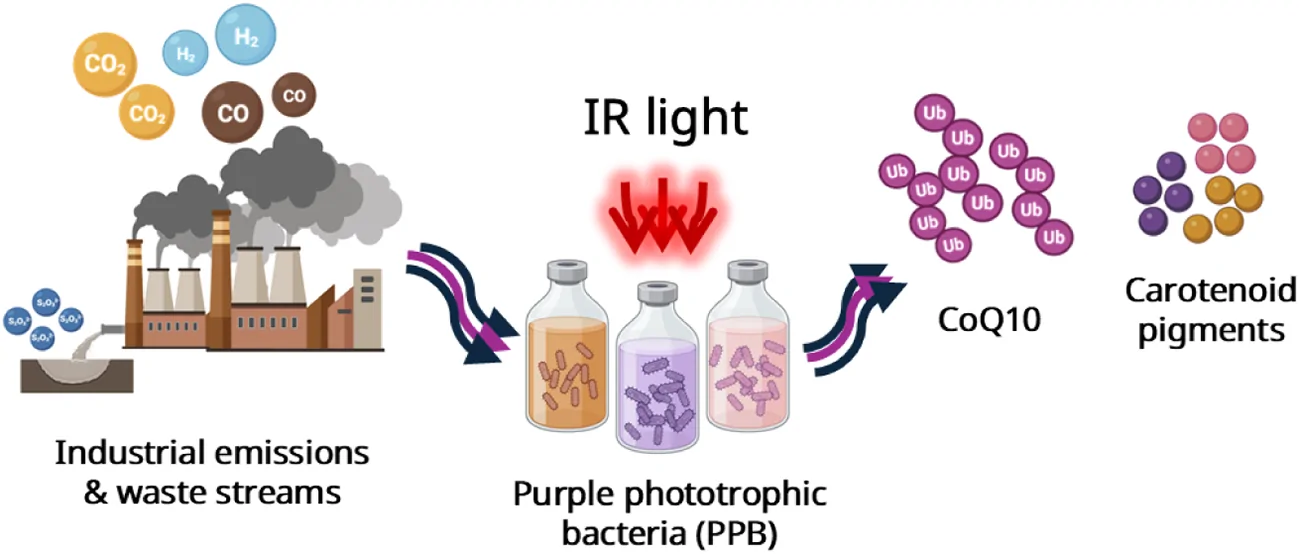

## 1 Introduction

The persistent increase in global waste generation, together with growing pressure to meet climate and waste-reduction targets, is driving a reassessment of carbon and energy management across industrial value chains.^1^ Despite ongoing decarbonization efforts, global CO_2_ emissions reached 37.8 Gt in 2024, with steel, cement and chemical sectors as major contributors.^2^ In parallel, industrial processes generate large quantities of reduced, energy-rich byproducts, including CO, H_2_ and sulfur species such as thiosulfate (S_2_O_3_^2–^), coemitted with CO_2_ or discharged through aqueous effluent streams.^3,4^ These streams are typically flared or oxidized for safety and regulatory compliance, generating additional CO_2_ and increasing operational costs, leaving up to 80–90% of industrial carbon and reducing power underutilized.^5,6^

Biotechnological systems that use CO_2_ as the sole carbon source while directly exploiting industrial reductants offer a promising route toward sustainable manufacturing. Such platforms could decouple production from sugar-based feedstocks and enable direct conversion of waste emissions into value-added chemicals, improving carbon valorization and process economics.^7^ However, integrating CO_2_ fixation with reduced industrial substrates remains challenging. Current gas fermentation systems are limited by low energy yields and gas–liquid mass transfer constraints,^8^ while mixotrophic approaches introduce additional carbon inputs that complicate mass balances and obscure the contribution of waste-derived reducing power.^9^ Additionally, most CO_2_-based bioprocesses target low-margin bulk products, whereas high-value metabolites may offer a more economically viable route for initial commercial deployment.^10,11^

Purple phototrophic bacteria (PPB) represent a promising yet underexplored platform for CO_2_ valorization. Using light as the primary energy source, PPB can fix CO_2_ while oxidizing a wide range of inorganic and organic electron donors. Unlike microalgae, PPB predominantly harvest near-infrared (IR) light, creating a distinct spectral niche that can be exploited through wavelength-selective illumination in closed photobioreactors. Their high carbon-use efficiency enables a large fraction of fixed CO_2_ to be directed toward biomass and intracellular products, minimizing carbon losses and making PPB particularly attractive for C1-based biomanufacturing. Moreover, their ability to sustain relatively high cell densities under comparatively low light intensities offers opportunities for cultivation in compact photobioreactors, potentially reducing the illuminated footprint, land requirements and water demand associated with conventional large-surface-area phototrophic systems. Independently, PPB generate ATP mainly through light-driven cyclic photophosphorylation, while electron donors provide the reducing equivalents required for CO_2_ fixation. This partial separation between energy conservation and reductant supply can alleviate some of the energetic constraints associated with chemolithotrophic gas fermentation.^12–14^ Moreover, PPB are intrinsically capable of synthesizing isoprenoid and quinone-derived metabolites, making them attractive platforms for converting CO_2_ into high-value bioactive compounds.

Among these, coenzyme Q10 (CoQ10) and carotenoids are particularly relevant. CoQ10 is a lipid-soluble quinone essential for cellular bioenergetics and widely commercialized as a nutraceutical due to its antioxidant and therapeutic properties.^15^ Its industrial production relies on heterotrophic fermentation with organic substrates, increasing costs and limiting sustainability.^16^ Carotenoids, including lycopene, β-carotene, spheroidene and spirilloxanthin, are hallmark PPB metabolites involved in light harvesting and photoprotection, with recognized antioxidant properties and broad applications in food, cosmetic and pharmaceutical industries.^17,18^

Despite these advantages, PPB research has largely focused on wastewater treatment, bioremediation and low-value products such as biomass and fertilizers.^19–21^ When industrial gases are considered, cultivation is typically heterotrophic or mixotrophic, requiring organic cosubstrates that complicate carbon balances and limit strict photoautotrophic CO_2_ conversion.^22,23^ Although PPB harbor pathways for CO and sulfur oxidation, their ability to use mixed industrial electron donors under autotrophic conditions while producing high-value metabolites remains largely unexplored.

Here, we demonstrate that PPB can sustain autotrophic growth on CO_2_ while utilizing industrially relevant electron donors, including H_2_, CO and S_2_O_3_^2–^, and simultaneously producing CoQ10 and carotenoid pigments. These findings establish PPB as a compelling platform for phototrophic CO_2_ valorization, linking carbon capture with the production of high-value pharmaceutical and nutraceutical compounds.

## 2 Materials and Methods

### 2.1 Selection of Potential PPB Able to Produce Coenzymes and Carotenoid Pigments from CO_2_

A targeted genome feature analysis based on Stegman et al.^24^ was conducted to identify PPB strains with metabolic potential for CO_2_ fixation, CO/S_2_O_3_^2–^ utilization and high-value metabolites biosynthesis. The original data set comprised 2,661 genomes covering 94 genera and was reduced to 1,348 genomes after quality filtering, duplicate removal and manual curation. For the present study, genomes were screened for the presence of key CO_2_ fixation pathways commonly found in PPB (Calvin–Benson–Bassham (CBB), reverse TCA (rTCA) and Wood–Ljungdahl), CO dehydrogenase (CODH) orthologs associated with CO oxidation (K01994, K00196, K00197, K00198 and K14138), and genes involved in CoQ10 and carotenoid biosynthesis (lycopene, β-carotene, spheroidene and spirilloxanthin). Reduced-sulfur oxidation capacity was additionally considered for PSB. Strains combining autotrophic potential, target-metabolite biosynthesis and different predicted electron-donor utilization capacities were further considered. Since these metabolic capacities were subsequently evaluated experimentally, strain availability from public culture collections was also included as a selection criterion. Based on these criteria, eight PPB strains available from the DSMZ culture collection were selected for experimental validation and subsequently classified as purple nonsulfur bacteria (PNSB) or purple sulfur bacteria (PSB) with S_2_O_3_^2–^ oxidation specifically considered within the PSB group.

The identified strains: *Rhodospirillum rubrum* DSM 467 and DSM 107, *Marichromatium purpuratum* DSM 1711, *Rhodovulum robiginosum* DSM 12329, *Rhodovulum euryhalinum* DSM 4868, *Rhodopseudomonas rhenobacensis* DSM 12706, *Rhodopseudomonas* sp. DSM 7375 and *Roseospira marina* DSM 15113, were acquired from the DSMZ culture collection (Leibniz-Institut, Germany). Actively growing strains were transferred to DSMZ-recommended media, whereas freeze-dried strains were activated following DSMZ protocols. Growth characteristics for each strain are provided in Supporting Information

### 2.2 Chemicals and Mineral Salt Medium

The autotrophic mineral medium was composed of (g L^–1^): MgSO_4_·7H_2_O-1.0, CaCl_2_·2H_2_O-0.11, NH_4_Cl-0.5, KNO_3_-1.0. Medium was supplemented with trace elements (mg L^–1^): CuCl_2_-0.01, FeCl_2_-0.9, ZnCl_2_-0.06, NiCl_2_-0.01, CoCl_2_-0.06, Na_2_MoO_4_-0.03, MnCl_2_-0.06, H_3_BO_3_-0.06, Na_2_SeO_3_-0.4, Na_2_WO_4_-0.01. NaCl was added during medium preparation at the optimum concentration for each PPB strain, ranging from 0.4 to 50 g L^–1^ (Supporting Information). l-cysteine monohydrate (0.3 g L^–1^) and resazurin solution (0.5 mL of 1 M) was added for anaerobic condition experiments. The medium was autoclaved at 1.5 atm at 121 °C for 20 min. After autoclaving, the medium was supplemented with filter-sterilized stock solutions of NaHCO_3_-2.5 (g L^–1^), K_2_HPO_4_ (0.5 g L^–1^) and vitamins using sterile 0.22 μm pore-size Millipore filters. The vitamin solution contained (mg L^–1^): biotin-0.02, nicotinamide-0.2, *p*-aminobenzoic acid-0.1, thiamine-0.2, pantothenic acid-0.1, pyridoxamine-0.5, cyanocobalamine-0.1 and riboflavin-0.1.

### 2.3 Experimental Setup

The experimental setup consisted of four sequential assays designed as a stepwise screening strategy (Figure 1). In Test 1, all strains identified from genome-featured analysis were evaluated for autotrophic growth using CO_2_ as the sole carbon source and H_2_ as electron donor based on biomass increase and substrate consumption. CoQ10 and carotenoid production were also analyzed to characterize the phototrophic metabolic potential of each strain, enabling the selection of the most promising candidates (*selected PPB strains*). In Test 2, the *selected PPB strains* were further evaluated under semicontinuous operation to assess autotrophic performance, growth stability, gas consumption and CoQ10 production over time. These strains were subsequently examined in Test 3 for their ability to grow autotrophically using alternative electron donors, specifically CO and S_2_O_3_^2–^, together with their capacity to produce CoQ10 and carotenoids. Finally, in Test 4, the best-performing strains under these conditions were evaluated under semicontinuous operation to compare substrate consumption dynamics, CoQ10 production and overall physiological performance.

**Figure 1:**
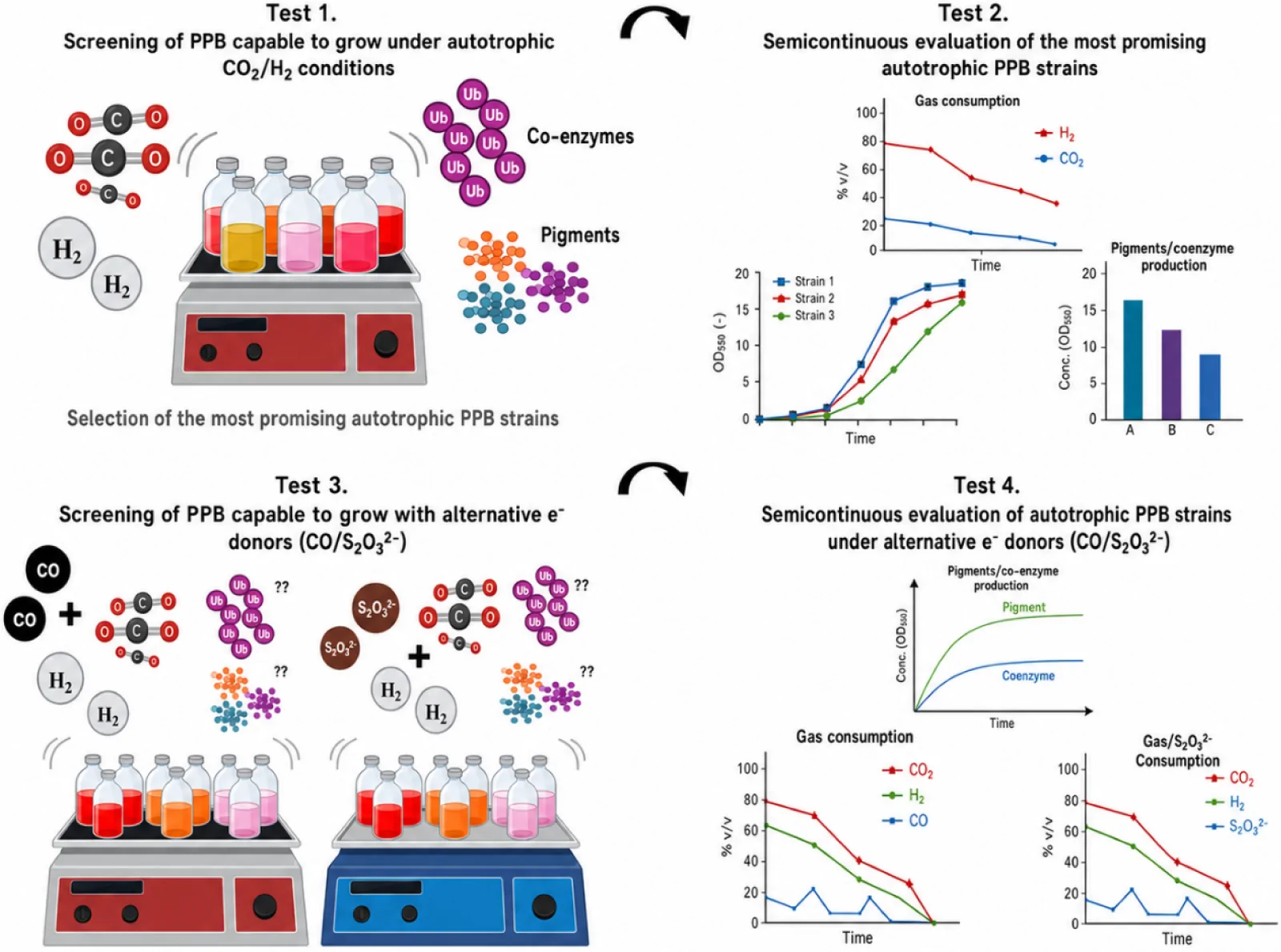
Schematic overview of the experimental design.

#### 2.3.1 Test 1: Laboratory Validation of Autotrophic Growth Using CO_2_ and H_2_

The eight PPB strains identified through the genome-guided analysis were initially cultivated in the corresponding DSMZ-recommended media and subsequently acclimated to autotrophic CO_2_/H_2_ conditions through three consecutive transfers in the autotrophic mineral medium. Acclimation was performed in triplicate 120 mL serum bottles containing 50 mL of autotrophic medium, adjusted to the strain-specific NaCl concentration indicated in Supporting Information. Bottles were closed with gastight butyl rubber septa and aluminum crimp caps and sterilized by autoclaving with their initial air headspace. After cooling to room temperature, the headspace was exchanged with a CO_2_/H_2_ mixture (20:80%, v/v) through sterile gas filters and adjusted to a final pressure of 1 atm. The bottles were subsequently inoculated with 1 mL of actively growing culture.

Cultures were incubated at 30 °C under orbital agitation at 140 rpm and continuous illumination provided by a 100 W tungsten-halogen lamp (Philips SA, Spain) positioned approximately 35 cm from the cultivation bottles. A broad-spectrum tungsten-halogen source was selected to provide a common illumination environment for the different PPB strains, covering wavelengths accessible to their carotenoid- and bacteriochlorophyll-containing photosynthetic systems without selecting a strain-specific narrow spectral band. The incident photon fluence rate at the culture position was 14.6 μmol photons m^–2^ s^–1^ over the 400–700 nm range, as measured using a spherical quantum sensor (US-SQS/L, Heinz Walz GmbH, Germany) connected to a LI-250A light meter (LI-COR Biosciences, USA). The lamp position and bottle arrangement were maintained constant throughout the experiments. Abiotic controls were prepared in parallel to confirm biotic gas consumption. Cultures were transferred three consecutive times under these conditions to ensure full adaptation to autotrophic growth. Actively growing cultures obtained after the third consecutive transfer were used as inocula for Test 2.

Headspace gas composition was monitored to quantify CO_2_ and H_2_ consumption, while liquid samples (5 mL) were periodically collected to determine biomass dry weight, pH, doubling time, CoQ10 and carotenoid production. Experiments were maintained until no further growth was observed.

#### 2.3.2 Test 2: Semicontinuous Evaluation of CO_2_ and H_2_ Consumption and CoQ10 Production

To further elucidate the metabolic behavior of the *selected PPB strains* under steady-state conditions, semicontinuous experiments were performed to evaluate gas consumption and CoQ10 production over time. Test 2 was inoculated with the actively growing CO_2_/H_2_-acclimated cultures obtained after the third consecutive transfer described in Test 1. Repeated-cycle semicontinuous experiments were conducted in triplicate 120 mL serum bottles under the same medium, temperature, agitation and illumination conditions described above. Three consecutive gas-consumption cycles were evaluated. The headspace was replaced when the gas concentrations decreased below 5% v/v and restored to the initial CO_2_/H_2_ composition of 20:80% v/v. Liquid samples (5 mL) were periodically collected to determine biomass concentration, CoQ10 content and pH. After sampling, fresh autotrophic medium was added to restore the initial working volume. Dissolved inorganic carbon was measured at the beginning and end of the experiment to determine total inorganic carbon consumption.

#### 2.3.3 Test 3: Assessment of CO and S_2_O_3_^2‑^ as Alternative Electron Donors in Autotrophic PPB Growth

Test 3 evaluated the ability of the *selected PPB strains* to sustain autotrophic growth using alternative electron donors, specifically CO and S_2_O_3_^2–^, and to support CoQ10 and carotenoid biosynthesis.

Experiments were conducted in triplicate using 120 mL bottles containing 50 mL of autotrophic medium prepared as described in Test 1. For CO-based growth, the headspace was adjusted to CO/CO_2_/H_2_ (10:18:72% v/v). This composition was selected as a simplified syngas-type mixture within the waste-gas valorization framework of the study, allowing the response of the selected PPB strains to CO to be evaluated while maintaining CO_2_ and H_2_ as components of the autotrophic gas mixture, whereas for S_2_O_3_^2–^-based growth, 10 mM S_2_O_3_^2–^ was added to the medium and the headspace adjusted to CO_2_/H_2_ (20:80% v/v). Exponentially growing autotrophic cultures (CO_2_/H_2_) were used as inoculum (5% v/v). CO- and H_2_ -containing gases were handled in a fume hood using gastight syringes and connections, and cultures were maintained in sealed serum bottles fitted with butyl septa throughout the experiments.

Cultures were incubated at the same conditions described previously. All conditions were tested in triplicate, including abiotic controls. CO consumption was monitored in the gas phase, whereas S_2_O_3_^2–^ consumption was measured in the liquid phase. Biomass and CoQ10 production were periodically monitored until no further growth was observed.

#### 2.3.4 Test 4: Semicontinuous Evaluation of Alternative Electron Donor Utilization

Semicontinuous experiments were conducted to evaluate substrate consumption dynamics and the production of pigments and coenzymes over time under alternative electron donor conditions (CO and S_2_O_3_^2–^). These experiments were performed using the strains that were able to grow on alternative electron donors in Test 3 (*R. rubrum* DSM 467 and *M. purpuratum*). Cultures were operated in semicontinuous mode in 120 mL bottles prepared under the previously described conditions.

Under CO-based conditions, three consecutive CO consumption cycles were evaluated. Similarly, under S_2_O_3_^2–^-based conditions, three consecutive consumption cycles were performed, with S_2_O_3_^2–^ initially supplied at 10 mM and resupplemented upon depletion to maintain consistent availability across cycles. Biomass, CoQ10, pH as well as DIC measurements and medium replenishment were conducted as described for Test 2.

### 2.4 Analytical Methods

#### 2.4.1 CO_2_, H_2_ and CO Monitorization

CO_2_, H_2_ and CO concentrations were quantified using an Agilent 7890A gas chromatograph equipped with a thermal conductivity detector (GC-TCD; Agilent Technologies, USA) and a CP Poraplot Q column (CP7554; 25 m × 0.53 mm × 20 μm). The oven, injector, and detector temperatures were set to 45, 150, and 200 °C, respectively, with helium as carrier gas at a flow rate of 13.7 mL min^–1^.

Aqueous CO_2_ was quantified as DIC using a TOC-VCSH analyzer (Shimadzu, Japan). Samples were filtered (0.22 μm) and adjusted to pH 9 with 0.5 M NaOH prior to analysis.

#### 2.4.2 Thiosulfate and Sulfate Quantification

S_2_O_3_^2–^ and sulfate (SO_4_^2–^) concentrations were quantified by ion chromatography using an HPLC-IC system (Waters, Bellefonte, USA) equipped with a Waters 432 conductivity detector and an IC-Pak Anion HC column (150 mm × 4.6 mm). Samples (1 mL) were filtered (0.22 μm), transferred to HPLC vials and stored frozen until analysis. Quantification was performed using external calibration curves prepared from standard solutions ranging from 0.1 to 25.0 g L^–1^.

#### 2.4.3 Ubiquinone (CoQ10) Extraction and Determination

CoQ10 extraction was performed by lyophilizing 2 mL biomass samples and resuspending them in 0.5 mL phosphate-buffered saline (PBS, 1×) containing 0.01 mM EDTA (pH 7.5). Cells were disrupted using zirconia beads in a bead beater (3 × 45 s cycles) under cold conditions. Subsequently, 0.5 mL of hexane:2-propanol (5:3 v/v) was added and samples were vortexed, shaken in the dark at low temperature for 30 min, and centrifuged (8,000 × g, 8 min). The upper organic phase containing CoQ10 was collected. All extraction steps were performed under cold and dark conditions to prevent degradation.

CoQ10 was analyzed by HPLC-DAD using a 717 Plus autosampler (Waters Alliance e2695, USA) coupled to a UV detector set at 275 nm. Separation was performed using an LC-18 AQ Supelcosil column with a C18 AQ precolumn (Sigma-Aldrich, Spain). The mobile phase consisted of water (A), acetonitrile (B), and ethyl acetate (C) with the following gradient: 0–10 min, 10% A, 90% B, 0% C; 10–25 min, 5% A, 60% B, 35% C; and 25–35 min, 5% A, 60% B, 35% C. The flow rate was 1 mL min^–1^, and the column temperature was maintained at 25 °C. CoQ10 was identified by comparison of its chromatographic retention with that of a commercial CoQ10 reference standard analyzed under identical conditions, together with detection at 275 nm, the characteristic absorption maximum of oxidized CoQ10. Quantification was performed by external calibration using the commercial CoQ10 standards (Sigma-Aldrich, Spain).

#### 2.4.4 Carotenoid Pigments Extraction and Identification

Carotenoid pigments were extracted by lyophilizing 2 mL biomass and resuspending it in an acetone: methanol mixture (7:3 v/v). Samples were vortexed, centrifuged (8,000 × g, 10 min) and the supernatant collected for analysis. The extraction was repeated until the pellet became colorless to ensure complete pigment recovery. All steps were performed under cold and dark conditions to prevent degradation.

Pigments were analyzed by HPLC-DAD using a 717 Plus autosampler (Waters Alliance e2695, USA) coupled to a UV dual-wavelength detector scanning 300–650 nm at 40 °C. Separation was performed using an LC-18 AQ Supelcosil column with a C18 AQ+ precolumn (Sigma-Aldrich, Spain). The mobile phase consisted of water (A), acetonitrile (B), and ethyl acetate (C), with the following gradient: 0–8 min, 6% A, 54% B, 40% C; 8–12 min, 10% A, 90% B; 12–14 min, 6% A, 54% B, 40% C. The flow rate was 1 mL min^–1^ and column temperature was set to 25 °C. Carotenoid pigments were qualitatively identified based on their chromatographic retention behavior and characteristic DAD absorption spectra by comparison with spectra reported in the literature and available databases.

#### 2.4.5 Biomass Determination

Biomass growth was monitored by measuring optical density at 650 nm (OD_650 nm_) using a SPECTROstar Nano spectrophotometer (BMG LABTECH, Germany). Dry biomass concentration was calculated as total suspended solids (TSS).

Doubling time (expressed in days) was calculated using eq 1, where G represents the generation time, t_1_ and t_2_ correspond to time 1 and time 2, and OD_1_ and OD_2_ correspond to the OD_650 nm_ values at t_1_ and t_2_, respectively.

1 $$G=\ln\left(2\right)\left(\frac{\left(t_{2}-t_{1}\right)}{\ln\left(\frac{OD_{2}}{OD_{1}}\right)}\right)$$

Medium pH was measured using a SensION+ pH3 pH meter (HACH, Spain).

#### 2.4.6 Statistical Analysis

Quantitative data are reported as mean ± standard deviation of three independent biological replicates (n = 3). Statistical analyses were performed using SPSS version 26.0 (IBM, USA). For comparisons among independent experimental groups, differences were evaluated by one-way analysis of variance (ANOVA) followed by Tukey’s HSD post hoc test for multiple comparisons. Homogeneity of variances was assessed using Levene’s test. Statistical significance was defined as *p* < 0.05.

## 3 Results and Discussion

### 3.1 Selection of Potential Autotrophic PPB for Metabolites Production

Although several PPB species have been reported to grow autotrophically using CO_2_, their capacity to simultaneously couple CO_2_ fixation with the biosynthesis of high-value metabolites remains largely unexplored. To address this gap, a genome-featured analysis was performed based on the data set of Stegman et al.,^24^ targeting PPB strains combining autotrophic potential with the capacity to produce CoQ10 and carotenoid pigments. From this analysis, strains were further selected based on their predicted electron donor utilization capacity and their availability as authenticated cultures for experimental validation. This resulted in the selection of eight PPB strains: *Rhodospirillum rubrum* DSM 467, *Rhodospirillum rubrum* DSM 107, *Marichromatium purpuratum* DSM 1711, *Rhodovulum robiginosum* DSM 12329, *Rhodovulum euryhalinum* DSM 4868, *Rhodopseudomonas rhenobacensis* DSM 12706, *Rhodopseudomonas* sp. DSM 7375 and *Roseospira marina* DSM 15113 (Figure 2).

**Figure 2:**
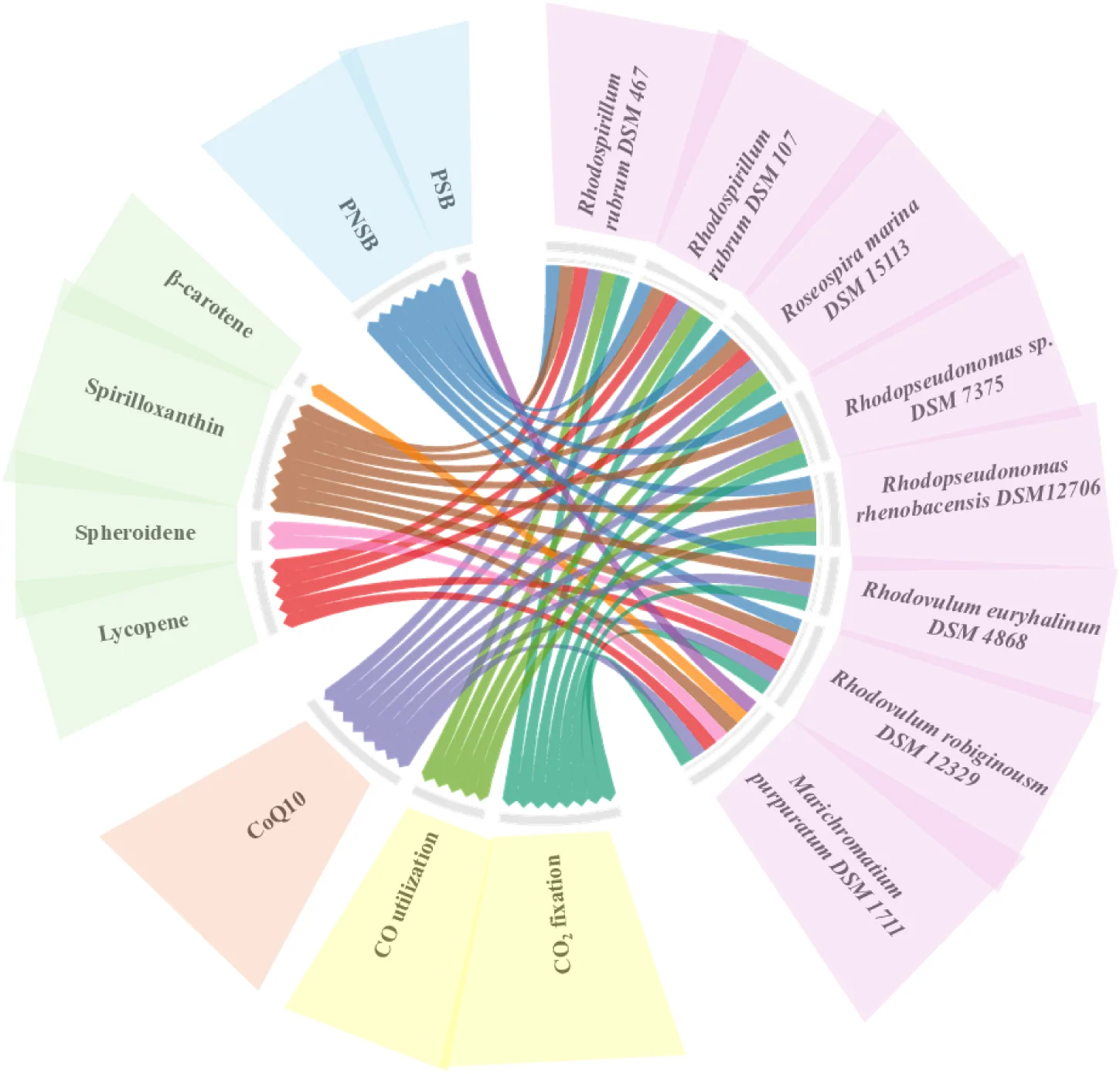
Genome-guided metabolic profile of the PPB strains selected for experimental validation. Chord diagram showing the distribution of genomic features associated with CO_2_ fixation, CO utilization and biosynthesis of high-value metabolites, including CoQ10 and carotenoids (lycopene, β-carotene, spheroidene and spirilloxanthin), across the selected strains. Connections indicate the presence of the corresponding genomic feature in each strain.

Beyond this shared metabolic core, clear functional differentiation emerged in their electron donor utilization potential. Several strains, including both *R. rubrum* strains, *Rhodopseudomonas* spp. and *R. marina*, encoded CODH orthologs, indicating the potential to oxidize CO. In contrast, *M. purpuratum* possessed genes associated with sulfur oxidation (e.g., *sox* pathway), consistent with its classification as a PSB, while strains such as *Rhodovulum* spp. retained autotrophic and biosynthetic pathways but lacked genomic capacity for CO utilization. This initial analysis identified a set of metabolically versatile candidates expected to differ primarily in electron acquisition, positioning them as promising candidates for autotrophic CO_2_ valorization into high-value metabolites.

### 3.2 Test 1: Laboratory Screening for Autotrophic Growth Using CO_2_ and H_2_

In Test 1, the eight PPB strains identified through genome feature analysis (Section 3.1) as potential autotrophic CO_2_ utilizers and metabolite producers were experimentally evaluated for their capacity to grow under CO_2_/H_2_ conditions. The average results obtained across replicates are summarized in Table 1. Although all strains encoded pathways associated with CO_2_ fixation, only six exhibited measurable autotrophic growth, indicating that the presence of these pathways does not necessarily translate into functional expression under the tested conditions.

*R. rubrum* DSM 467 and DSM 107 exhibited the shortest doubling times and reached biomass concentrations of approximately 0.5 g L^–1^ (1.03 ± 0.11 d and 1.18 ± 0.20 d, respectively. This behavior is consistent with their known metabolic flexibility, which enables efficient redox balancing and coupling between H_2_ oxidation and CO_2_ fixation.^25,26^ However, growth remained slower than under photoheterotrophic conditions (∼0.5 d), reflecting the higher energetic demand of autotrophic metabolism.^27^ Both strains produced CoQ10 and spheroidene/spirilloxanthin-type carotenoids, with characteristic absorption peaks at ∼430/456/488 nm and ∼468/496/530 nm,^28^ confirming that secondary metabolism remains active under these conditions. In *R. rubrum*, CoQ10 exhibits a characteristic absorption maximum at 275 nm, whereas related quinones show distinguishable spectral characteristics. For example, rhodoquinone and reduced CoQ10 exhibited maxima at approximately 284 and 292 nm, respectively.^29^
*S. purpuratum* also sustained autotrophic growth, reaching a biomass concentration of 0.50 ± 0.02 g L^–1^ and displaying a longer doubling time of 2.73 ± 0.24 d. CoQ10 was detected, in agreement with previous observations in metabolically versatile phototrophic *Proteobacteria*.^30^ Pigment analysis identified okenone as the dominant carotenoid based on its characteristic absorption around 487 nm.^31^ As a pigment specific to *Chromatiaceae*, okenone is associated with enhanced light harvesting under low-light conditions, reflecting adaptation to stratified aquatic environments.^32^

**Table 1:** Autotrophic Growth Performance and Metabolite Profiles of the Purple Phototrophic Bacterial Strains Screened under CO_2_/H_2_ Conditions^a^

Strain	Autotrophic growth under CO_2_/H_2_	Doubling time (d)	Maximum biomass (g L^–1^)	Biomass productivity (g L^–1^ d^–1^)	CoQ10 detected	Carotenoid(s) detected
****R. rubrum**** **467**	Yes	1.03 ± 0.11	0.51 ± 0.05	0.16 ± 0.08	Yes	Spheroidene/spirilloxanthin-like
****R. rubrum**** **107**	Yes	1.18 ± 0.20	0.53 ± 0.09	0.10 ± 0.01	Yes	Spheroidene/spirilloxanthin-like
****R. robiginosum****	Yes	1.09 ± 0.18	0.32 ± 0.10	0.10 ± 0.04	No	Spheroidene-like
****M. purpuratum****	Yes	2.73 ± 0.24	0.50 ± 0.02	0.06 ± 0.01	Yes	Okenone
****R. marina****	Yes	7.72 ± 0.88	0.33 ± 0.13	0.02 ± 0.01	No	Spirilloxanthin-like
****Rhodopseudomonas**** **sp.**	Yes	15.11 ± 1.61	0.25 ± 0.10	0.01 ± 0.01	Yes	Spheroidene-like
****R. rhenobacensis****	No	N.D.	N.D.	N.D.	N.D.	N.D.
****R. euryhalinum****	No	N.D.	N.D.	N.D.	N.D.	N.D.

a Values are expressed as mean ± standard deviation of three independent biological replicates (*n* = 3). N.D., not determined due to the absence of autotrophic growth. Carotenoid assignments were qualitative and based on chromatographic retention behavior and characteristic DAD absorption spectra compared with literature and database spectra. Structurally related carotenoids, geometric isomers or coeluting compounds cannot be unequivocally distinguished without further structural confirmation.

In contrast, *R. robiginosum* exhibited rapid initial growth (1.09 ± 0.18 d) but lower final biomass accumulation (0.32 ± 0.10 g L^–1^), in accordance with its specialization in phototrophic Fe^2+^ oxidation, where ferrous iron acts as the primary electron donor.^33^ Under the tested conditions, trace Fe^2+^ likely supported early growth, while its depletion constrained further biomass accumulation. CoQ10 was not detected, which may reflect a strain-specific quinone composition, a comparatively small CoQ10 pool or a concentration below the analytical detection limit. Carotenoid profiles were dominated by spheroidene-type pigments, in agreement with previous observations in *Rhodovulum* species.^34^

*R. marina* and *Rhodopseudomonas* sp. exhibited significantly slower growth, with doubling times of 7.72 ± 0.88 d and 15.11 ± 1.61 d, respectively, indicating strong energetic constraints under CO_2_/H_2_ conditions. The ability of *R. marina* to sustain autotrophic growth contrasts with previous studies reporting no detectable growth under comparable conditions and suggests that expression of its autotrophic metabolism is highly condition dependent.^35^ Only weak CoQ10 detection was observed in *R. marina*, indicating a comparatively small detectable CoQ10 pool under the tested conditions. Similarly, the low growth rate of *Rhodopseudomonas* sp. likely reflected strain-specific constraints rather than genus-wide limitations, as other members of this genus have exhibited efficient growth under chemolithoautotrophic conditions.^36^ In this strain, CoQ10 was detected and carotenoid analysis confirmed spheroidene-type pigments, consistent with the dominant biosynthetic pathway in PNSB.^37^

Based on these results, *R. rubrum* DSM 467 and DSM 107, together with *M. purpuratum*, were selected for further evaluation due to their combined autotrophic performance and metabolite production capacity.

### 3.3 Test 2: Semicontinuous Evaluation of CO_2_/H_2_ Consumption and CoQ10 Production

In Test 2, semicontinuous operation was used to assess CO_2_ and H_2_ consumption and the stability of CoQ10 production during repeated gas replacement cycles in *R. rubrum* DSM 467, *R. rubrum* DSM 107 and *M. purpuratum* (Figure 3).

**Figure 3:**
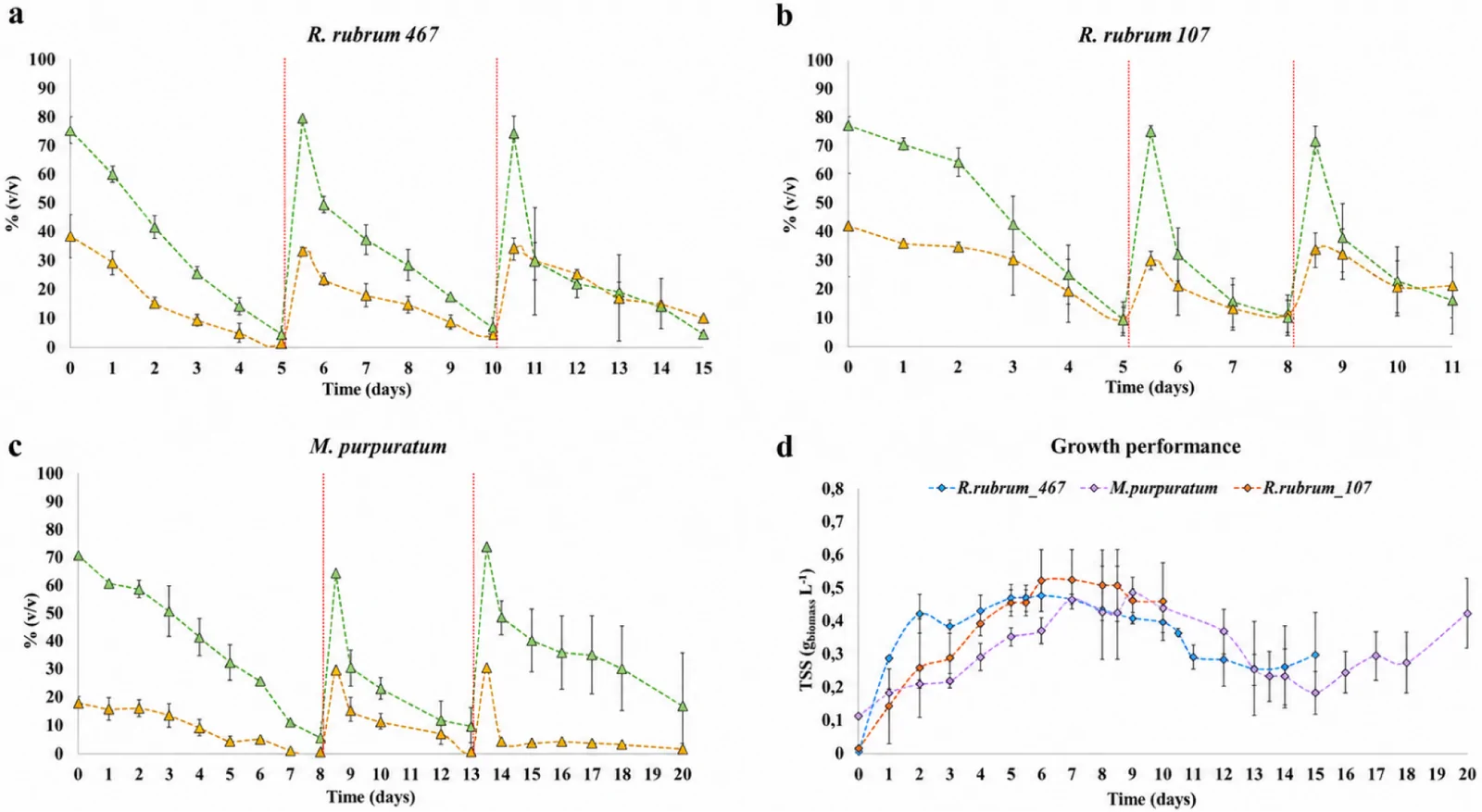
CO_2_ and H_2_ concentrations (% v/v) during semicontinuous autotrophic cultivation of (a) *R. rubrum* DSM 467, (b) *R. rubrum* DSM 107 and (c) *M. purpuratum*. CO_2_ (▲ yellow dashed line) and H_2_ (▲ green dashed line). Gas replacements are indicated by red vertical dashed lines. (d) Biomass concentrations for *R. rubrum* DSM 467 (⧫ blue dashed line), DSM 107 (⧫ orange dashed line) and *M. purpuratum* (⧫ purple dashed line). Data are presented as mean ± SD of three independent cultures (*n* = 3).

Both *R. rubrum* strains exhibited comparable biomass-specific CO_2_ consumption of 2.20 ± 0.21 and 2.34 ± 0.15 gCO_2_ g_biomass_^–1^ for DSM 467 and DSM 107, respectively (Figure 3a and Figure 3b), corresponding to apparent biomass yields of 0.455 and 0.427 g_biomass_ gCO_2_^–1^ (Supporting Information). These values remained stable throughout the experiment, indicating sustained autotrophic carbon fixation despite gradual limitation in gas availability. However, from the third gas replacement cycle (day 11), biomass concentration in *R. rubrum* DSM 467 decreased from 0.44 ± 0.1 g L^–1^ to 0.24 ± 0.15 g L^–1^. This decline occurred together with substantial pH fluctuations during the CO_2_ consumption cycles. Culture pH increased from approximately 7.5 at the beginning of cultivation to 7.9–8.1 during the first cycle and reached 8.2–8.5 during later cultivation. This pattern suggests that the bicarbonate/phosphate buffering capacity of the medium was insufficient to maintain stable pH under prolonged autotrophic operation, with the highest pH values coinciding with reduced biomass accumulation. Despite this, CO_2_ consumption remained stable, indicating that carbon fixation was not inhibited. This sustained activity is consistent with the metabolic flexibility of *R. rubrum*, where carbon assimilation is primarily driven by the CBB cycle via RubisCO form II, while H_2_ oxidation supplies the reducing power required for CO_2_ reduction and biomass synthesis.^38,39^

*M. purpuratum* (Figure 3c) showed a biomass-specific CO_2_ consumption of 1.80 ± 0.50 gCO_2_ g_biomass_^–1^, corresponding to an apparent biomass yield of 0.556 g_biomass_ gCO_2_^–1^ (Supporting Information). From the second gas replacement cycle onward, biomass decreased significantly from 0.51 ± 0.10 to 0.22 ± 0.12 g L^–1^, before recovering after day 15 to 0.43 ± 0.22 g L^–1^. Despite these biomass fluctuations, CO_2_ consumption remained stable, indicating sustained autotrophic carbon fixation. This transient decline was therefore attributed to physiological adaptation or biomass aggregation affecting biomass quantification, rather than to a loss of metabolic activity, as supported by the subsequent recovery. Genomic evidence further supports this interpretation, as *M. purpuratum* encodes RubisCO, consistent with an active CBB cycle.^40^

All three strains showed net H_2_ consumption although not at levels typically associated with obligate hydrogenotrophs,^41^ with values of 4.37 ± 0.3 × 10^–3^, 4.20 ± 0.3 × 10^–3^ and 2.85 ± 0.5 × 10^–3^ gH_2_ g_biomass_^–1^ for *R. rubrum* DSM 467, *R. rubrum* DSM 107 and *M. purpuratum*, respectively. Since *R. rubrum* is known to produce H_2_ via nitrogenase under nitrogen-limiting phototrophic conditions, a possible contribution of endogenous H_2_ production was considered. However, nitrogenase activity in *R. rubrum* has been reported to be strongly repressed at millimolar ammonium concentrations.^42^ In the present study, the medium contained approximately 9.35 mM NH_4_^+^ together with 9.89 mM NO_3_^–^, making nitrogenase-mediated H_2_ production highly unlikely under the nitrogen-replete conditions applied. Therefore, the observed profiles were interpreted as net H_2_ consumption, although low-level simultaneous H_2_ production cannot be completely excluded.

CoQ10 production differed strongly among strains (Figure 4). *R. rubrum* DSM 467 reached 5.9 ± 0.2 mg_CoQ10_ g_biomass_^–1^, whereas DSM 107 accumulated significantly higher levels (9.61 ± 0.2 mg_CoQ10_ g_biomass_^–1^, *p* < 0.05) under autotrophic CO_2_/H_2_ conditions. In contrast, *M. purpuratum* produced only 0.2 ± 0.1 mg_CoQ10_ g_biomass_^–1^. The substantial pH variations observed during prolonged cultivation may also have contributed to differences in CoQ10 accumulation by affecting cellular physiology. Notably, CoQ10 levels in *R. rubrum* exceed most reported values for natural PPB under photoheterotrophic or mixotrophic conditions, including *Rhodopseudomonas palustris* (2.0–3.0 mg_CoQ10_ g_biomass_^–1^),^43^ *Rhodobacter sphaeroides* (3.7–5.4 mg_CoQ10_ g_biomass_^–1^)^44^ and previously reported *R. rubrum* strains (2.3–3.0 mg_CoQ10_ g_biomass_^–1^).^45^ Furthermore, CoQ10 levels in DSM 107 approach those reported for engineered *R. sphaeroides* HY01 strains (11.5–13.3 mg_CoQ10_ g_biomass_^–1^).^46^ Together, these results demonstrated the strong intrinsic capacity of *R. rubrum* for CoQ10 production under autotrophic conditions, with DSM 107 emerging as a particularly promising candidate for sugar-free biomanufacturing. Further structural confirmation by mass spectrometry would nevertheless strengthen the discrimination of CoQ10 from closely related quinones.^47^

**Figure 4:**
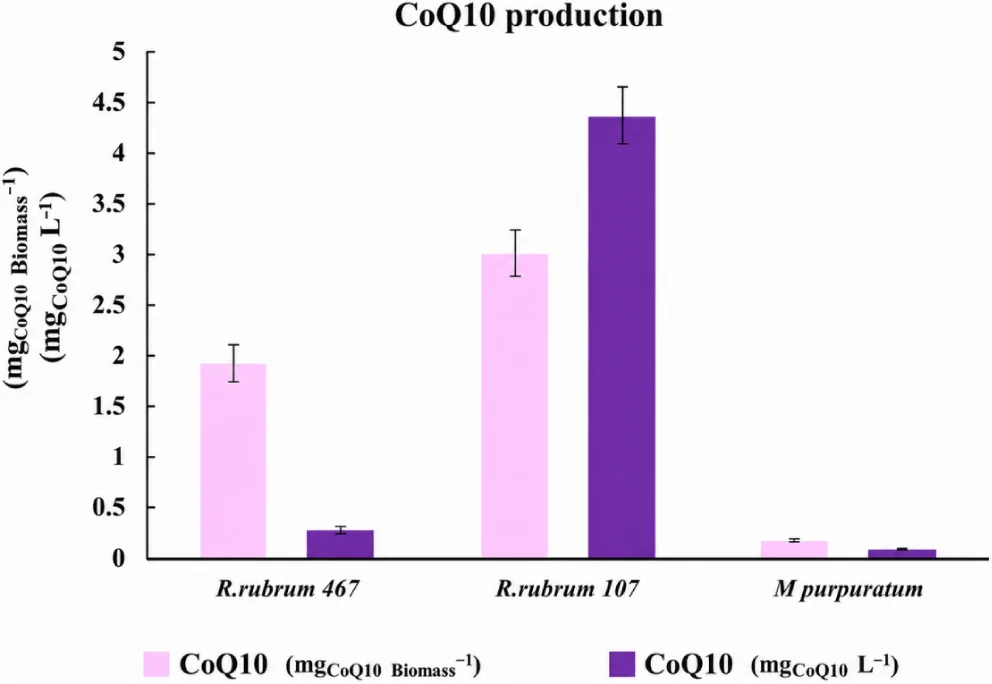
CoQ10 production in *R. rubrum* DSM 467, *R. rubrum* DSM 107 and *M. purpuratum* under autotrophic CO_2_/H_2_ conditions shown as specific content (pink bars, mg_CoQ10_ g_biomass_^–1^) and volumetric concentration (purple bars, mg_CoQ10_ L^–1^). Data are presented as mean ± SD of three independent cultures (*n* = 3).

### 3.4 Test 3: Screening of Alternative Electron Donors: CO-Containing (Syngas) and Thiosulfate Containing Conditions

In Test 3, *R. rubrum* DSM 467, *R. rubrum* DSM 107 and *M. purpuratum* were evaluated for their ability to utilize alternative electron donors, specifically CO (CO/CO_2_/H_2_ gas mixture) and S_2_O_3_^2–^ (S_2_O_3_^2–^ and CO_2_/H_2_ gas mixture). Genome-featured analysis (Section 3.1) indicated that both *R. rubrum* strains encode CODH genes associated with CO oxidation, whereas *M. purpuratum*, as a PSB, harbored sulfur oxidation pathways. Accordingly, all three strains were tested under both conditions to assess electron donor utilization, tolerance and effects on growth (Table 2).

**Table 2:** Effect of Alternative Electron Donors on Growth and Substrate Conversion^a^

Strain	Condition (electron donor)	Doubling time (d)	Biomass-specific substrate consumption	Metabolite production	Byproduct formation	Observations
*R. rubrum* DSM 467	CO/CO_2_/H_2_	1.50 ± 0.60	0.04 ± 0.01 gCO g _biomass_^–1^	CoQ10 + spheroidene/spirilloxanthin-like pigments	Net H_2_ production observed	Active CO oxidation. Water-gas shift activity
*R. rubrum* DSM 107	CO/CO_2_/H_2_	1.45 ± 0.50	CO consumption not detected	N.D	—	CO tolerated. No measurable CO oxidation
*M. purpuratum*	CO/CO_2_/H_2_	12.90 ± 0.71	CO consumption not detected	N.D	—	CO inhibits autotrophic growth
*R. rubrum* DSM 467	H_2_/CO_2_ + S_2_O_3_^2–^	1.48 ± 0.20	S_2_O_3_^2–^ consumption not detected	N.D	—	S_2_O_3_^2–^ not utilized. No inhibition
*R. rubrum* DSM 107	H_2_/CO_2_ + S_2_O_3_^2–^	1.62 ± 0.30	S_2_O_3_^2–^ consumption not detected	N.D	—	S_2_O_3_^2–^ not utilized. No inhibition
*M. purpuratum*	H_2_/CO_2_ + S_2_O_3_^2–^	1.12 ± 0.30	2.88 mmol S_2_O_3_^2–^ g_biomass_^–1^	CoQ10 + okenone	5.76 mmol SO_4_^2–^ g_biomass_^–1^	Sox oxidation pathway. Dual electron donor usage

a Values represent mean ± standard deviation of biological replicates. N.D. not determined.

Under CO-containing conditions, *R. rubrum* DSM 467 and DSM 107 showed comparable doubling times (1.50 ± 0.60 and 1.45 ± 0.50 d, respectively; *p* > 0.05). However, their response to CO differed markedly. DSM 467 actively oxidized CO (0.04 ± 0.01 gCO g_biomass_^–1^) while simultaneously producing H_2_, consistent with an active water–gas shift metabolism.^48^ In contrast, no detectable CO consumption was observed in DSM 107 despite sustained growth in the CO/CO_2_/H_2_ mixture, indicating that CO was tolerated but did not measurably contribute as an electron donor under the tested conditions. Since both strains encode CODH-associated genes, these results highlight that genomic potential does not necessarily translate into measurable CO oxidation and suggest strain-specific control of CO metabolism.^48^ As expected, *M. purpuratum* did not oxidize CO, and its presence strongly inhibited autotrophic growth, increasing the doubling time nearly 6-fold (12.9 ± 0.7 d). This behavior suggests CO toxicity or interference with key enzymatic reactions or electron transfer processes.^49^

Under S_2_O_3_^2–^ amended conditions, neither *R. rubrum* strain exhibited detectable S_2_O_3_^2–^ utilization, consistent with the absence of active sulfur oxidation pathways. Importantly, S_2_O_3_^2–^ did not inhibit growth, with doubling times of 1.48 ± 0.20 d and 1.62 ± 0.30 d for DSM 467 and DSM 107, respectively, comparable to S_2_O_3_^2–^-free conditions. In contrast, *M. purpuratum* efficiently utilized S_2_O_3_^2–^ as an additional electron donor, exhibiting a doubling time of 1.12 ± 0.30 d and a biomass-specific consumption of 2.88 mmol S_2_O_3_^2–^ g_biomass_^–1^, coupled with SO_4_^2–^ production (5.76 mmol SO_4_^2–^ g_biomass_^–1^). Notably, this strain simultaneously oxidized H_2_ and S_2_O_3_^2–^, indicating parallel electron input into the photosynthetic electron transport chain. This dual electron donor utilization likely enhanced reducing power and ATP generation, explaining the improved growth relative to CO_2_/H_2_ alone (2.73 ± 0.24 d versus 1.12 ± 0.30 d).

HPLC-DAD analysis showed that both *R. rubrum* DSM 467 and *M. purpuratum* maintained their qualitative CoQ10 and carotenoid profiles under alternative electron donor conditions. In *R. rubrum* DSM 467, CoQ10 production and spheroidene/spirilloxanthin-type carotenoids were preserved under CO-driven conditions, while *M. purpuratum* maintained CoQ10 and okenone production under S_2_O_3_^2–^ conditions. The persistence of CoQ10 and carotenoids under light conditions is consistent with maintenance of the photosynthetic electron-transfer apparatus. CoQ10 is not considered a specific component of the Coo-dependent CO-oxidation pathway. Therefore, under CO-containing conditions, cyclic photophosphorylation may operate alongside CO-dependent energy conservation.^50,51^

### 3.5 Test 4: Semicontinuous Operation under Alternative Electron Donors: Substrate Utilization and CoQ10 Production

In Test 4, semicontinuous cultivation was performed to evaluate CO-driven syngas utilization by *R. rubrum* DSM 467 and S_2_O_3_^2–^-driven growth by *M. purpuratum*, together with CoQ10 production over time (Figure 5).

**Figure 5:**
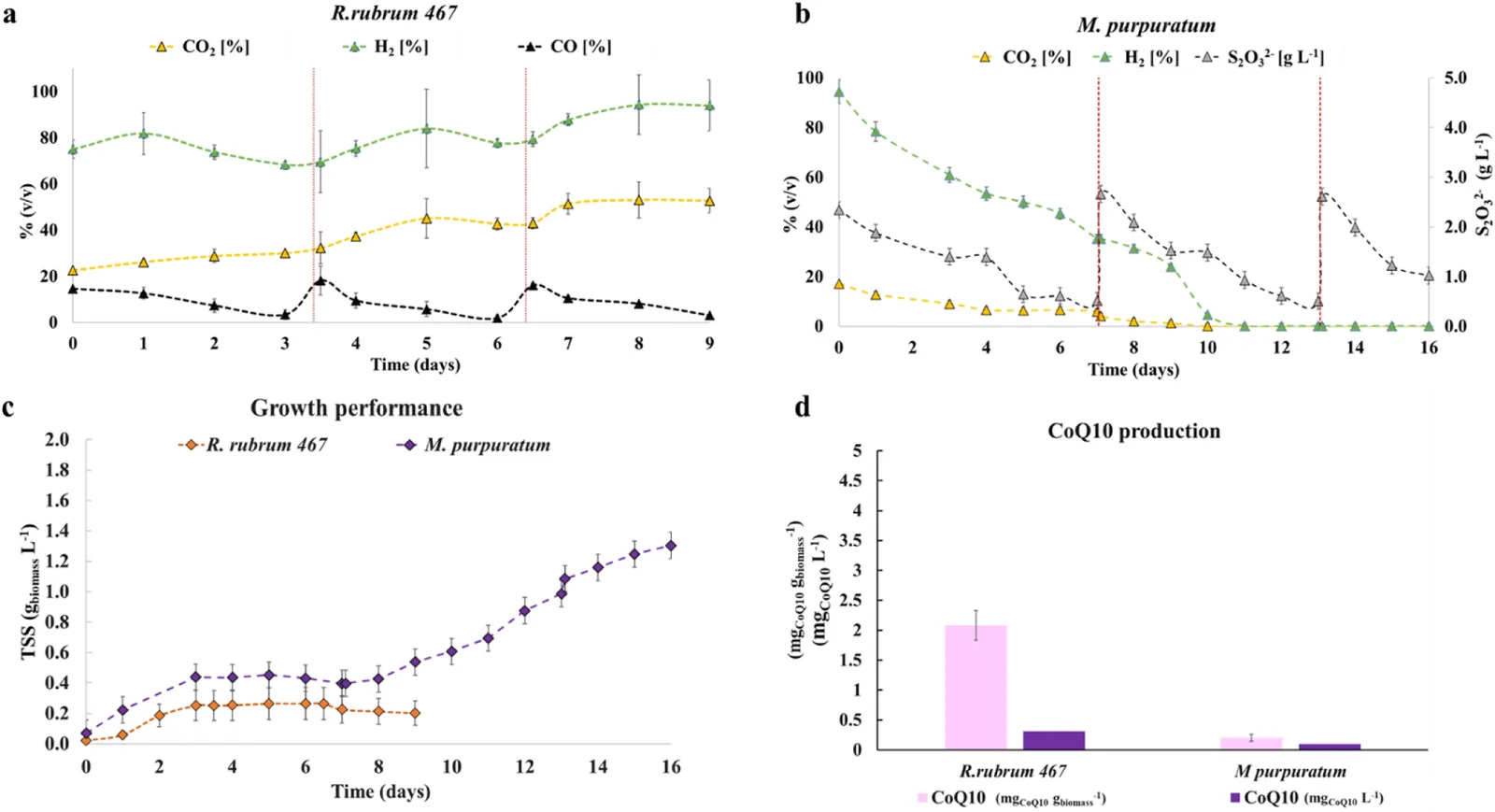
Substrate concentration during semicontinuous autotrophic cultivation of (a) *R. rubrum* DSM 467 (CO/CO_2_/H_2_) and (b) *M. purpuratum* (S_2_O_3_^2–^/CO_2_/H_2_): CO (▲ black dashed line), CO_2_ (▲ yellow dashed line), H_2_ (▲ green dashed line) and S_2_O_3_^2–^ (▲ gray dashed line). Gas/S_2_O_3_^2–^ replacements are indicated by red vertical dashed lines. (c) Biomass concentration (TSS, g L^–1^) for *R. rubrum* DSM 467 (⧫ orange dashed line) and *M. purpuratum* (⧫ purple dashed line). (d) CoQ10 production under respective conditions: specific content (pink bars, mg_CoQ10_ g_biomass_^–1^) and volumetric concentration (purple bars, mg_CoQ10_ L^–1^). Data are presented as mean ± SD of three independent cultures (*n* = 3).

Under CO-containing conditions, *R. rubrum* DSM 467 showed repeated CO depletion following headspace replenishment, accompanied by changes in the H_2_ and CO_2_ profiles (Figure 5a). Across the experiment, average CO consumption was 0.43 ± 0.11 gCO g_biomass_^–1^, with net H_2_ production of 0.05 ± 0.03 gH_2_ g_biomass_^–1^. A theoretical gas-availability assessment showed that, immediately after replenishment, the initial molar amount of H_2_ in the headspace was approximately 7.2-fold higher than that of CO. The estimated equilibrium dissolved concentration of H_2_ was also substantially higher than that of CO (0.553 versus 0.091 mmol L^–1^; Supporting Information) Thus, the observed CO-conversion pattern cannot be attributed to lower initial H_2_ availability following headspace replenishment.

The observed CO consumption together with net H_2_ formation is consistent with the well-established Coo-dependent water–gas shift pathway of *R. rubrum*. In this system, CO oxidation by CODH supplies electrons through CooF to the CO-induced hydrogenase, resulting in proton reduction and H_2_ formation.^52^ Importantly, the membrane-associated Coo system has been associated with CO-dependent energy conservation independently of light, as demonstrated by the ability of *R. rubrum* to grow on CO under anaerobic dark conditions.^52^ Under the light conditions used in this study, CO-dependent energy conservation could therefore operate alongside cyclic photophosphorylation, although their relative contributions cannot be distinguished from the present measurements.

The H_2_ generated through the Coo pathway may additionally be available for reoxidation through the distinct uptake-hydrogenase system of *R. rubrum* (Hup), providing a potential route for recycling CO-derived reducing equivalents. The observed net accumulation of H_2_ during CO conversion nevertheless indicates that H_2_ formation exceeded its overall consumption at the culture level. As CO availability decreased, the accompanying changes in the H_2_ and CO_2_ profiles were compatible with an increasing contribution of H_2_-supported phototrophic metabolism under light, in which H_2_ can provide reducing equivalents while light-driven photophosphorylation supplies cellular energy.

In contrast, *M. purpuratum* (Figure 5b) simultaneously utilized H_2_ and S_2_O_3_^2–^. During the initial phase (days 0–9), concurrent H_2_ oxidation (0.13 ± 0.06 gH_2_ g_biomass_^–1^) and S_2_O_3_^2–^consumption (0.28 ± 0.08 gS_2_O_3_^2–^ g_biomass_^–1^), together with SO_4_^2–^ production (0.78 ± 0.24 gSO_4_^2–^ g_biomass_^–1^), confirmed dual-donor oxidation. Following H_2_ depletion (day 11), biomass-specific S_2_O_3_^2–^consumption increased (0.50 ± 0.12 gS_2_O_3_^2–^ g_biomass_^–1^), accompanied by higher SO_4_^2–^ production (1.10 ± 0.30 gSO_4_^2–^ g_biomass_^–1^), indicating a shift toward sulfur-driven metabolism. Available genomic evidence for *M. purpuratum* (GenBank assembly accession GCA_000224005.3) supports both a NAD-linked Hox hydrogenase and a membrane-associated uptake hydrogenase, providing potential routes for H_2_-derived electrons toward the pyridine-nucleotide and quinone pools, respectively. S_2_O_3_^2–^ oxidation is instead supported by SoxAX/B/YZ together with sulfur-storage and reverse-Dsr machinery, followed by SoeABC-mediated oxidation of sulfite to sulfate. The presence of DsrL further provides a potential connection between sulfur oxidation and pyridine-nucleotide reduction. In addition, TsdBA represents an alternative S_2_O_3_^2–^-oxidation route that has been experimentally linked to HiPIP and the photosynthetic reaction center in *M. purpuratum*.^53^ Thus, light-driven cyclic electron transport could primarily support ATP generation, whereas H_2_- and sulfur-derived electrons can enter distinct redox pools that ultimately contribute to the reducing requirements of autotrophic CO_2_ fixation.

CoQ10 production differed significantly between strains (Figure 5d). In *R. rubrum* DSM 467, the specific CoQ10 content under CO-driven conditions decreased to 2.1 ± 0.5 mg_CoQ10_ g_biomass_^–1^, approximately 3-fold lower than under CO_2_/H_2_ conditions (5.9 ± 0.2 mg_CoQ10_ g_biomass_^–1^), despite sustained CO oxidation. One mechanistically plausible interpretation is that this decrease reflects remodeling of the cellular quinone pool in response to the different electron-transfer regime imposed by CO metabolism. *R. rubrum* is known to synthesize both CoQ10 and rhodoquinone, with rhodoquinone providing a lower-potential quinone pool suited to highly reducing electron-transfer reactions.^54^ Thus, redistribution between CoQ10- and rhodoquinone-containing pools could contribute to the lower CoQ10 accumulation observed under CO-containing condition. This interpretation is consistent with the known quinone flexibility of *R. rubrum*, although condition-dependent responses, including changes in quinone biosynthesis or photosynthetic membrane composition, may also contribute to the observed decrease in CoQ10.^55^ Further characterization of the quinone pool would be necessary to clarify the relative contribution of these mechanisms.

In contrast, *M. purpuratum* maintained a stable CoQ10 content (0.2 ± 0.1 mg_CoQ10_ g_biomass_^–1^), consistent with its role as a tightly regulated structural component of the electron transport chain. Under these conditions, excess reducing power appeared to be preferentially directed toward biomass formation rather than increased quinone synthesis (Figure 5c).^56^

Taken together, these findings provide a laboratory-scale proof of concept that PPB can couple autotrophic CO_2_ fixation with the strain-specific utilization of industrially relevant electron donors for high-value metabolite production. However, translation to larger-scale photobioreactors will require optimization of gas–liquid mass transfer, light distribution and penetration, reactor geometry, mixing and process control. In particular, the low solubility of CO and H_2_ and the attenuation of NIR light at high cell densities represent key constraints for process intensification, highlighting the need for reactor configurations that combine efficient gas-transfer zones with independently controlled illuminated sections. These engineering advances should be complemented by genome-scale metabolic modeling and metabolic flux analysis to identify metabolic bottlenecks and optimize carbon and electron partitioning toward target products. Continuous and intensified cultivation, including reduced-light or dark-production strategies where feasible, should also be explored to increase volumetric productivity, while long-term operational stability, safe handling of CO- and H_2_-containing gas streams, sustainable downstream recovery and techno-economic and life-cycle assessments will ultimately determine the industrial feasibility of PPB-based C1 bioprocesses.^57,58^

## Supplementary Material



## Data Availability

The raw and source data supporting the findings of this study have been deposited in the Zenodo repository and are publicly available at 10.5281/zenodo.22643023.

## Notes

Growth characteristics of the selected PPB strains; theoretical carbon-allocation analysis and apparent biomass yield during semicontinuous CO_2_/H_2_cultivation; theoretical assessment of gas availability and CO mass-transfer requirements during semicontinuous cultivation of *R. rubrum* DSM 467 (PDF)
