# Supplementary material for: Purple Phototrophic Bacteria as a Novel Platform for Coenzyme Q10 Production from CO2, Syngas, and Sulfur Waste Byproducts
Source: Environ Sci Technol. 2026 Oct 22;60(44):31733–44. doi: 10.1021/acs.est.6c07111 (PMC13757935; doi:10.1021/acs.est.6c07111)
Supplement: Supplementary file 1 [file es6c07111_si_001.pdf]

Supporting Information

Purple phototrophic bacteria as a novel platform for coenzyme Q10 production from CO_2, syngas and sulfur waste byproducts

Elisa Huang Lin[ab], Raquel Lebrero[ab], Sara Cantera[ab]*

[a] Department of Chemical Engineering and Environmental Technology, University of Valladolid, Dr. Mergelina s/n., Valladolid, 47011, Spain

[b] Institute of Sustainable Processes, University of Valladolid, Dr. Mergelina s/n., Valladolid, 47011, Spain

*Corresponding author: Sara Cantera; email: sara.cantera@uva.es

Table S1. Growth characteristics of the selected PPB strains.

PPB strain	Taxonomic group	Family	Salinity % NaCl (optimum)	Temperature (optimum)	DSMZ medium	pH (optimum)
Rhodovulum robiginosum DSM 12329	Alphaproteobacteria	Paracoccaceae	1.0–3.0%	25–35 °C	929	7.0–7.5
Rhodovulum euryhalinum DSM 4868	Alphaproteobacteria	Paracoccaceae	2.0–5.0%	25–35 °C	28 + 3% NaCl	7.0–7.5
Rhodospirillum rubrum DSM 467	Alphaproteobacteria	Rhodospirillaceae	<1.0%	28–35 °C	27	6.8–7.2
Rhodopseudomonas rhenobacensis DSM 12706	Alphaproteobacteria	Nitrobacteraceae	0.5–2.0%	25–35 °C	26	5.0–8.0
Roseospira marina DSM 15113	Alphaproteobacteria	Rhodospirillaceae	1.0–3.0%	28–32 °C	998	7.5–8.0
Marichromatium purpuratum DSM 1711	Gammaproteobacteria	Chromatiaceae	1.0–5.0%	30–37 °C	28 + 5% NaCl + 0.1% thiosulfate	7.0–7.5
Rhodopseudomonas sp. DSM 7375	Alphaproteobacteria	Nitrobacteraceae	0.5–2.0%	25–35 °C	27	6.8–7.2
Rhodospirillum rubrum DSM 107	Alphaproteobacteria	Rhodospirillaceae	<1.0%	30–35 °C	27	6.8–7.2

S2. Theoretical carbon-allocation analysis and apparent biomass yield relative to CO_2 consumption in Test 2.

Table S2. Theoretical carbon allocation and apparent biomass yield during semicontinuous autotrophic CO_2 /H_2 cultivation.

Strain	Biomass-specific CO_2 consumption (g CO_2 g biomass^{-1})	CO_2 -C consumed (g C g biomass^{-1})	Assumed biomass-C (g C g biomass^{-1})	Apparent biomass-C recovery (%)	Apparent biomass yield, YX/CO_2 (g biomass g CO_2^{-1})
Rhodospirillum rubrum DSM 467	2.20 ± 0.21	0.600 ± 0.057	0.488	81.3	0.455
Rhodospirillum rubrum DSM 107	2.34 ± 0.15	0.638 ± 0.041	0.488	76.4	0.427
Marichromatium purpuratum	1.80 ± 0.50	0.491 ± 0.136	0.488	99.4	0.556

Biomass-specific CO_2 consumption values are reported as mean ± standard deviation. Biomass carbon was estimated assuming the empirical biomass composition $CH_{1.8}O_{0.5}N_{0.2}$, corresponding to 0.488 g C g biomass^{-1}.

S3. Theoretical assessment of gas availability and CO mass-transfer requirements

S3.1 Experimental basis and initial gas availability

Semicontinuous cultivation of *Rhodospirillum rubrum* DSM 467 was performed in 120 mL serum bottles containing 50 mL of liquid medium, corresponding to an estimated headspace volume of 70 mL. Following gas replenishment, the headspace composition was adjusted to $CO/CO_2/H_2$ at 10:18:72% (v/v) at 1 atm and cultures were incubated at 30 °C and 140 rpm. Initial gas inventories were estimated using the ideal gas law:

$$n_i = \frac{y_i\, P\, V_G}{RT}$$

where n_i is the amount of gas i (mol), y_i is its molar fraction in the headspace, P is the total pressure (atm), V_G is the headspace volume (L), R is the ideal gas constant (0.082057 L atm mol^{-1} K^{-1}), and T is the absolute temperature (K).

Based on these conditions, the initial headspace contained approximately 0.281 mmol CO and 2.026 mmol H_2, corresponding to an H_2/CO molar inventory ratio of 7.2.

S3.2 Theoretical dissolved-gas availability

The theoretical equilibrium dissolved concentrations of CO and H_2 were estimated from Henry's law:

$$C_i = K_H, iP_i$$

where Ci is the equilibrium dissolved concentration and Pi is the corresponding gas partial pressure. At 30 °C, the calculated initial equilibrium concentrations were approximately 0.093 mmol L^{-1} for CO and 0.554 mmol L^{-1} for H_2.

S3.3 Lower-bound CO mass-transfer requirement

To assess whether the observed CO disappearance required unusually high gas-transfer capacity, a theoretical lower-bound requirement was calculated. The average volumetric CO disappearance rate was estimated from the initial CO inventory, the 50 mL working volume and the approximately 2.5-3.0 d required for CO to approach depletion:

$$rCO = kLa\,(C^*{}_{co} - C_{co},L)$$

Assuming $C_{co},L \approx 0$ provides the maximum possible concentration driving force and therefore the minimum required to support the average observed CO disappearance:

Gas-liquid transfer can be expressed as:

$$KLa^{req} = \frac{rCO}{C^*{}_{co}}$$

yielding a theoretical lower-bound requirement of approximately 0.85 -1.02 h^{-1}.

Table S3. Summary of gas availability and theoretical CO mass-transfer requirements during semicontinuous cultivation of *Rhodospirillum rubrum* DSM 467

Parameters	Value
Initial CO amount in the headspace	0.281 mmol
Initial H_2 amount in the headspace	2.026 mmol
Initial H_2 /CO molar ratio in the headspace	7.20
Equilibrium dissolved CO concentration,	0.091 mmol L^{-1}
Equilibrium dissolved H_2 concentration,	0.553 mmol L^{-1}
Total initial CO amount	0.286 mmol
Total initial H_2 amount	2.054 mmol
Total H_2 /CO molar ratio	7.18
Average volumetric CO disappearance rate	0.079–0.095 mmol L^{-1} h^{-1}
Minimum theoretical requirement	0.87-1.04 h^{-1}

S3.4 Interpretation

The theoretical assessment showed that H_2 was not initially less available than CO. Following headspace replenishment, the H_2 inventory was approximately 7.2-fold higher than that of CO, and its estimated equilibrium dissolved concentration was also substantially higher. Therefore, the observed CO-conversion pattern cannot be attributed to a lower initial availability of H_2
